# Implications of Oxidative and Nitrosative Post-Translational Modifications in Therapeutic Strategies against Reperfusion Damage

**DOI:** 10.3390/antiox10050749

**Published:** 2021-05-08

**Authors:** Mabel Buelna-Chontal, Wylly R. García-Niño, Alejandro Silva-Palacios, Cristina Enríquez-Cortina, Cecilia Zazueta

**Affiliations:** Department of Cardiovascular Biomedicine, National Institute of Cardiology Ignacio Chávez, Mexico City 14080, Mexico; mabel.buelna@comunidad.unam.mx (M.B.-C.); ramses.garcia@cardiologia.org.mx (W.R.G.-N.); alejandro.silva@cardiologia.org.mx (A.S.-P.); dracristinaenriquez@hotmail.com (C.E.-C.)

**Keywords:** oxidative post-translational modification, nitrosative post-translational modification, reperfusion damage, mitochondria

## Abstract

Post-translational modifications based on redox reactions “switch on-off” the biological activity of different downstream targets, modifying a myriad of processes and providing an efficient mechanism for signaling regulation in physiological and pathological conditions. Such modifications depend on the generation of redox components, such as reactive oxygen species and nitric oxide. Therefore, as the oxidative or nitrosative milieu prevailing in the reperfused heart is determinant for protective signaling, in this review we defined the impact of redox-based post-translational modifications resulting from either oxidative/nitrosative signaling or oxidative/nitrosative stress that occurs during reperfusion damage. The role that cardioprotective conditioning strategies have had to establish that such changes occur at different subcellular levels, particularly in mitochondria, is also presented. Another section is devoted to the possible mechanism of signal delivering of modified proteins. Finally, we discuss the possible efficacy of redox-based therapeutic strategies against reperfusion damage.

## 1. Introduction

**Figure 1:**
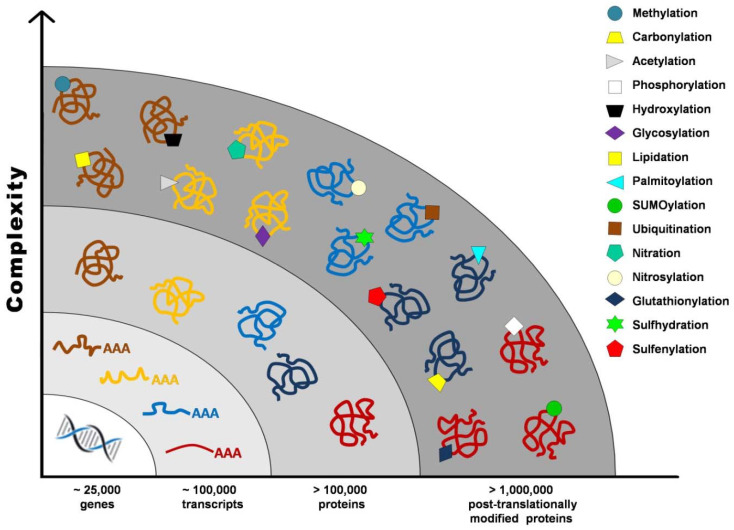
Post-translational modifications increase proteome complexity.

Proteome once defined as the “protein complement of the genome” cannot be accurately established without taking into account post-translational modifications (PTMs), such as proteolysis, phosphorylation, glycosylation, glutathionylation, S-nitrosylation, and carbonylation, that contribute to the vast number of gene products from a single transcript. It has been estimated that the human genome contains between 20,000 and 25,000 genes, whereas the transcriptome is composed of around 100,000 transcripts, and the proteome exceeds 1 million proteins (Figure 1) [1].

PTM of proteins based on redox reactions is a sophisticated and efficient mechanism conserved throughout evolution, which regulates intracellular signaling. Such modifications change the biological activity of downstream targets, modifying cellular processes such as autophagy [2], the immune and antioxidant response [3,4], energy metabolism [5], protein folding and degradation [6], proliferation and differentiation [7,8], and also affect the activity of transcription factors through direct and indirect mechanisms [9].

Reactive oxygen species (ROS) and reactive nitrogen species (RNS) interact with redox-sensitive signaling molecules that contain cysteine residues, forming disulfide cross-links, producing nitration of tyrosine residues by S-nitrosylation [10] or carbonylation of specific amino acid residues [11]. Besides, the typical examples of PTMs, i.e., phosphorylation and dephosphorylation, are also regulated by redox-based mechanisms. Tyrosine phosphatases contain reactive and redox-sensitive cysteine residues located in its active site that, being deprotonated at physiological pH, exist as a thiolate anion (Cys–S−). Oxidation of this residue to a sulfenic acid by hydrogen peroxide (H_2_O_2_) inactivates the tyrosine phosphatases; such inactivation becomes irreversible after the addition of two molecules of sulfinic acid or of three molecules of sulfonic acid [12]. Therefore, ROS/RNS inhibit the activity of tyrosine phosphatases and increase tyrosine phosphorylation. In addition, reactive cysteines in protein kinases are directly modified by ROS/RNS, resulting in further protein activation. A good example is mitogen-activated protein kinases (MAPKs), which play a central role in coupling extracellular signals to different biological processes such as gene expression, proliferation, differentiation, and cell death [13].

Another relevant aspect is that ROS/RNS are not only considered essential signaling molecules, but sustained high levels of these reactive species cause intracellular damage in several pathologies. Therefore, this review article focuses on redox PTMs triggered by ROS/RNS in cardiac reperfusion damage and their role on cardioprotective signaling at different subcellular levels, particularly in mitochondrial function. Finally, we discuss the possible efficacy of redox-based therapeutic strategies against reperfusion damage.

## 2. Oxidative and Nitrosative Stress

Oxidative stress is the imbalance between the production and elimination of ROS produced by the cells. These species include a wide variety of molecules and radicals derived from molecular oxygen (O_2_), which are considered physiological byproducts of mitochondrial oxidative metabolism [14]. O_2_ can be reduced by one electron at a time, producing relatively stable intermediates, such as superoxide anion (O_2_^•−^), that can be spontaneously or enzymatically dismutated to H_2_O_2._ This reactive specie can diffuse freely across cellular membranes, acting as a signaling molecule through the oxidation of target proteins that activate signaling cascades of transducers such as ion transporters, receptors, kinases, phosphatases, and transcription factors [15]. In turn, H_2_O_2_ can be fully reduced to water or partially reduced to hydroxyl (OH•) radicals, in a reaction catalyzed by transition metals.

There are different possible sources of ROS in the myocardium such as xanthine oxidoreductase, NADPH oxidoreductases, cyclooxygenases, the mitochondrial electron transport chain, and neutrophil activation [16]. NADPH oxidases are membrane-bound proteins that catalyze the electron transport from NADPH to molecular oxygen [17]. The NOX family comprises seven homologues: NOX1, NOX2, NOX3, and NOX5, which mainly produce superoxide, as well as NOX4, NOX6/DUOX1, and NOX7/DUOX2 that generate H_2_O_2_ [18]. Remarkably, NOX1, NOX2, NOX4, and NOX5 are expressed in the cardiovascular system, and their increased activation correlates positively with hypertension, atherosclerosis, ischemia/reperfusion (IR) injury, hypertrophy, and coronary artery disease [19]. However, it has been recognized that NOX4 exerts protection in the cardiovascular system; for example, endothelial-specific overexpression of NOX4 enhances vasodilatation and decreases blood pressure through the generation of peroxide hydrogen and the prevention of superoxide-mediated NO inactivation [20]. Besides, NOX4 limits infarct size in hearts subjected to IR [21] and promotes angiogenesis in response to cardiac adaptation to overload stress [22].

Mitochondria are considered the main source of ROS in mammalian cells and, according to the redox-optimized ROS balance hypothesis, are overproduced either when the mitochondrial redox state is highy reduced or highly oxidized. In reduced conditions, a slow electron flow favors their reaction with molecular O_2_, independently of the antioxidant capacity; on the other hand, when the redox balance shifts towards an oxidized state, the scaveging activity diminishes, promoting ROS overflow [23]. In terms of physiological signaling, this hypothesis provides an explanation of maximal energy output with minimal ROS production, as well as maximal ROS production at unbalanced redox states in pathological conditions.

Superoxide-producing domains in Complex I (CI) are the flavin mononucleotide (FMN) domain in the NADH-binding site (site IF) and the coenzyme Q (CoQ) binding site [13,18]. As mentioned, ROS generation is favored in the IF domain when electron transport occurs under highly reductive conditions, i.e., high proton motive force (Δp) and low rate of ATP synthesis (state 4). On the other hand, the inhibition of the CoQ-binding site by rotenone triggers O_2_^•−^ production, drawing back electrons onto FMN, generating more O_2_^•−^. Even more, reverse electron transport (RET), induced by a highly reduced state of the CoQ pool in combination with mitochondrial hyperpolarization (inactive complex V), forces electrons back from CoQH2 into CI, thus reducing NAD+ to NADH at the FMN and producing ROS. The site of ROS production by RET has not been determined, but it is proposed to be the flavin site of CI, the ubiquinone-binding site of CI, or the iron-sulphur cluster [24]. RET is thought to be the major source of O_2_^•−^ generation during both physiological and patological conditions. Chouchani et al. (2014) demonstrated that fumarate increases during ischemia and is reduced to succinate via reversal of succinate dehydrogenase. Accumulated succinate is oxidized during reperfusion, activating conventional electron transport through complexes III and IV, but also enhancing RET at CI, producing ROS that contribute to IR injury [25]. Complex III has also been pointed out as a source of O_2_^•−^; however, this only happens if O_2_ reacts with a ubisemiquinone bound to the Qo site, when mitochondria are supplied with CoQH_2_ and the Qi site is inhibited by antimycin [26]. Under physiological conditions, O_2_^•−^ production is lower and negligible as compared with maximum rates produced by CI during RET [27]; however, damage to CI during IR may induce the loss of protein–protein interaction between this complex and Complex III, enhancing ROS production by Complex III [28]. Besides the respiratory chain, other proteins are potential ROS sources in mitochondria. In this respect, monoamine oxidase B (MAO-B) augments H_2_O_2_ production, as it was detected with mitochondrial specific probes in neonatal and adult WT cardiomyocytes and compared with those lacking MAO-B [29]; also, the cytosolic adaptor protein p66^Shc^, which is translocated to mitochondria in response to stress, catalyzes electron transfer from cytochrome c to O_2_ and has been causally related to reperfusion damage due to mitochondrial ROS formation [30]. Finally, the nicotinamide nucleotide transhydrogenase (NNT), indispensable for maintaining NADPH availability, might be linked to mitochondrial ROS production, as it is required for the peroxidase activities that removes H_2_O_2._, resulting from excess substrate availability [31]. However, experimental evidence of NNT-mediated ROS removal is scarce, and more studies in situ or in vivo are necessary to demonstrate that its activity is physiologically coupled with mitochondrial respiration.

The essential role of ROS/RNS in cellular signaling processes has led to developing the concept of oxidative eustress (Greek eu, meaning good, well, positive), whereas the supra-physiological levels of ROS/RNS are proposed to be named oxidative distress, i.e., unspecific oxidative reactions leading to cellular damage [32]. That is, when the ROS threshold surpasses the equilibrium maintained by the antioxidant system, the cell will be in distress. In this sense, it is relevant to mention that mitochondrial antioxidant machinery includes systems that regulate and control H_2_O_2_ depending on the availability of NADPH. Mitochondria express two NADPH-dependent antioxidant systems: GSH and TRX2. The oxidation of two molecules of reduced glutathione (GSH) and one molecule of water forms glutathione disulfide (GSSG) by the activity of the glutathione peroxidase (GPX1/4). GSH reestablishment depends on the glutathione reductase (GR) that depends on NADPH [33]. On the other hand, mitochondria consume H_2_O_2_ in a respiration-dependent way via the thioredoxin/peroxiredoxin (Trx/Prx) system. The contribution of both antioxidant systems was demonstrated with the generation of the *Trx2* gene, knockout (KO) mice that resulted in embryonic lethality, while the Gpx1 and Gpx4 gene KO only increased mitochondrial sensitivity against ROS.

The equilibrium between the antioxidant response and redox homeostasis is relevant for the hormetic response of mitochondria. This is an adaptative state that results from a gradual increase or exposure to low ROS levels, which promotes signaling pathways that dimish cell susceptibility to subsequent stress [34]. This adaptative mechanism, named mito-hormesis, is associated with the above-mentioned concept of oxidative eustress, that might be taken into account to avoid hindering a normal hormetic response, by indiscriminately applying antioxidant therapies.

O_2_^•−^ reacts with other radicals such as NO, producing peroxynitrite (ONOO^−^), which, along with other oxidants derived from NO, are referred to as RNS [35]. NO, a highly reactive molecule with astonishingly diverse roles in human physiology and disease, was first described by Furchgott and colleagues more than 20 years ago [36]. NO modulates cardiac function by regulating vascular tone [37], excitation–contraction coupling [38], platelet aggregability [39], and mitochondrial function [40]. NO is produced after L-arginine oxidation by the three isoforms of the nitric oxide synthase (NOS) [41]. Endothelial NOS (eNOS) is localized in the caveolae of the sarcolemma and neuronal NOS (nNOS) in the sarcoplasmic reticulum [42]. Both are constitutively expressed, whereas inducible NOS (iNOS) is only expressed under stress conditions, usually in pro-inflammatory states [43]. Nowadays, it is well established that NO and their derivatives act as key signaling molecules in cardioprotection through the activation of soluble guanylate cyclase (sGC) to synthesize the intracellular second messenger cyclic guanosine monophosphate (cGMP), which targets ion channels, cGMP-regulated phosphodiesterases, and cGMP-dependent protein kinases [44]. Another mechanism involves the direct protein S-nitrosylation, in which a NO moiety is covalently attached to the free thiol of a cysteine residue to modify the activity of target proteins [45].

### Switch of Oxidative/Nitrosative Signaling to Deleterious Oxidative/Nitrosative/Nitrative Stress

As described, RNS and ROS are involved in signaling events; nonetheless, when over-generated and/or deregulated, they can lead to oxidative stress and eventually to cell death. Cells have developed systems to fight against ROS and their deleterious effects. Then, the control of ROS generation relies on the relative rates of electron reaction with oxygen and the activity of antioxidants present in the matrix and the cytoplasm [16]. The amount of ROS to switch between redox signaling and oxidative stress remains unknown, due to the difficulty of measuring O_2_^•−^ and H_2_O_2_ production in vivo. Although some estimates have been made, these are only approximate under particular experimental conditions [27].

The rate of electron leak depends mainly on the redox state of a particular carrier. The estimated electron leak in the mETC is 0.1–0.5% under homeostatic conditions for skeletal muscle; this rate changes if more substrates are supplied and there is more ATP synthesis. Thus, the leaking rate depends on the type of substrates and the respiratory state, where state 3 resembles more closely the physiological in vivo levels [16].

Early studies have demonstrated that endogenously produced NO can reduce oxidative stress, terminate free radical chain reactions within the lipid membrane, and attenuate inflammatory response [46]. Its critical role in protecting the myocardium from IR injury has been well established [47]. At a very low concentration (pM), NO induces the activation of high-affinity primary binding targets, but if this concentration is overpassed (50–300 nM), the response could be either beneficial (wound/healing repair) or detrimental (oncogenesis). Nitrative and nitrosative stress is defined by a NO concentration higher than 1 μM [48].

## 3. Mechanistic Clues of eNOS/NO/SNO Cardioprotective Signaling

Ischemia-reperfusion injury occurs when blood supply to an organ is disrupted and then restored by removing the obstruction. Irreversible myocardial damage occurs after prolonged and severe ischemia, during myocardial infarction and stroke, but also in transplantation [49]. Current treatments for myocardial ischemia include surgical treatments such as percutaneous coronary intervention and coronary artery bypass graft, as well as drug therapies [50]. Although reperfusion of ischemic tissue is essential for survival and represents the most favorable therapeutic strategy to prevent IR injury, the restoration of blood flow initiates pathological events leading to cell death through the exacerbated generation of mitochondrial ROS and RNS [51]. Specifically, excessive and unregulated NO synthesis by NOS has been implicated as causal or a contributor to reperfusion damage, associated with the toxicity of NO, ONOO^−^, and their derived species, which favor polynitrosylation and oxidation of cysteine thiols, as well as nitration of tyrosine in proteins [52]. In conjunction, oxidative and nitrosative stress damage biological membranes and cellular components when the antioxidant capacity is exceeded, resulting in cardiomyocyte injury, cardiac contractile dysfunction, arrhythmias, and endothelial dysfunction [53].

The first evidence of the relevance of eNOS/NO/ S-nitrosylation (SNO) in cardioprotective signaling came from several studies using distinct approaches, such as eNOS or iNOS transgenic models, pharmacological inhibitors of NOS activity, as well as NO precursors and NO donor treatments. Regarding transgenic models, it was demonstrated that deletion of the eNOS gene exacerbates IR injury in mice [54]; however, Kanno et al. (2000) observed that NO increases in eNOS^−^^/^^−^ hearts, through the compensatory induction of iNOS producing myocardial protection [55]. In contrast, genetic overexpression of eNOS in mice, and specifically in cardiomyocytes, attenuates myocardial infarction, and such protection is lost when NOS is inhibited with N^G^-nitro-L-arginine methyl ester (L-NAME) [56]. Besides, constitutive overexpression of iNOS in transgenic mice protects against IR injury by preventing mitochondrial permeability transition pore (mPTP) opening [57].

On the other hand, the pharmacological inhibition of NOS exacerbates IR injury in the heart [55,58], whereas NO precursors such as L-arginine and L-citrulline attenuate IR injury [59]. In addition, NO donors including S-nitrosoglutathione (GSNO) [60], glyceryl trinitrate (GTN) [61], nitrite [62], or paramagnetic nanoparticles coated with S-nitrosothiols [63] prevent IR injury. For example, the NO donor S-nitroso-N-acetylpenicillamine (SNAP) reduces the infarct area and preserves contractile function in isolated perfused mice hearts [55], while the NO donor NOC-18 induces changes in the mitochondrial phosphoproteome and prevents mPTP and apoptosis triggering after ischemia, probably through the activation of protein kinase C (PKC) or protein kinase G (PKG) [64]. Besides, the infusion of the NO donors such as 3-morpholinosydnonimine-*N*-ethylcarbamide (SIN-1) and isosorbide dinitrate, previous to IR, promotes SNO formation in rat heart membranes [65]. Román-Anguiano et al. (2019) demonstrated that SNAP and the 17β-aminoestrogen compound Prolame, which induces NO production, prevent cardiac reperfusion damage and improve functional recovery in post-ischemic hearts by activating both cGMP-dependent and SNO pathways [66]. Contradictorily, there is evidence showing that NO production may be harmful for cardiomyocytes under IR conditions, and the discrepancies in the published data as well as the potential confounding factors that affect experimental results on the role of NO in myocardial IR damage are not discussed here, but are reviewed in depth elsewhere [67].

**Figure 2:**
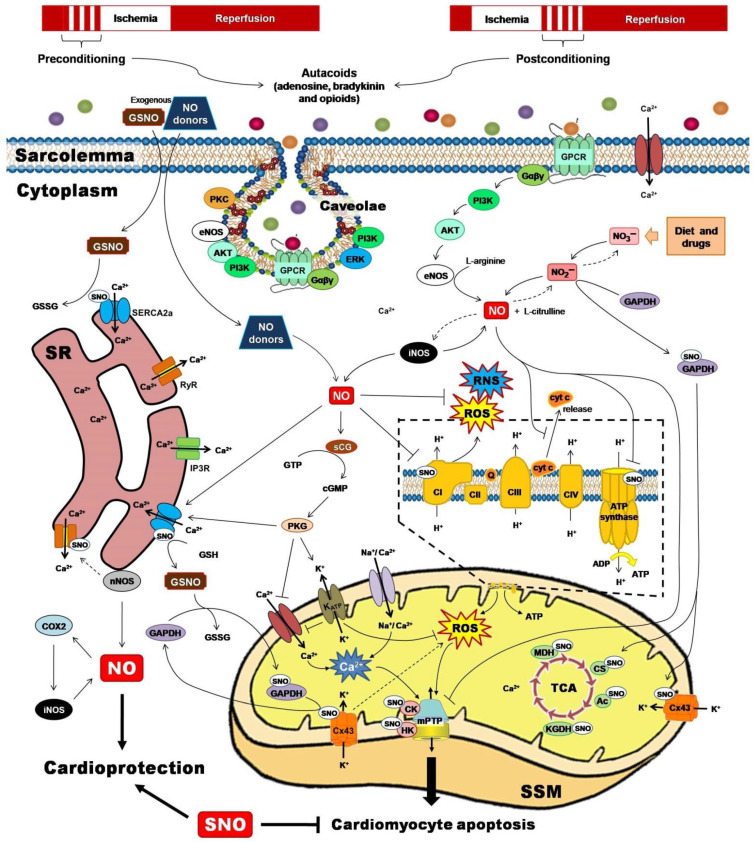
Ischemic preconditioning and ischemic postconditioning confer cardioprotection by activating nitric oxide synthase (NOS)/nitric oxide (NO)/S-nitrosylation-mediated pathways. NO and their derivatives reduce the initial burst of reactive oxygen species (ROS) and reactive nitrogen species (RNS), activate redox signaling, and trigger protective mechanisms or adaptive responses that culminate at the mitochondrial level, inhibiting Complex I-induced ROS generation, reducing adenosine triphosphate (ATP) synthesis, preventing calcium (Ca^2+^) overload, blocking the permeability transition pore opening (mPTP), and lowering cytochrome c (cyt C) release. Ac, aconitase; AKT, serine/threonine kinase; CI-CIV, complexes I to IV; cGMP, cyclic guanosine monophosphate; CK, creatine kinase; COX2, cyclooxygenase-2; CS, citrate synthase; Cx43, connexin 43; eNOS, endothelial nitric oxide synthase; ERK, extracellular signal-regulated kinase; Gαβγ, G proteins; GAPDH, glyceraldehyde-3-phosphate dehydrogenase; GPCR, G protein-coupled receptor; GSH, reduced glutathione; GSNO, S-nitrosoglutathione; GSSG, oxidized glutathione; GTP, guanosine triphosphate; HK, hexokinase; iNOS, inducible nitric oxide synthase; IP_3_R, inositol 1,4,5-trisphosphate receptor; K_ATP_, ATP-sensitive potassium channel; KGDH, α-ketoglutarate dehydrogenase; MDH, malate dehydrogenase; nNOS, neuronal nitric oxide synthase; PI3K, phosphatidylinositol 3-kinase; PKC, protein kinase C; PKG, protein kinase G; Q, ubiquinone; RyR, ryanodine receptor; SERCA2a, sarcoplasmic reticulum Ca^2+^-ATPase; sGC, soluble guanylate cyclase; SR, sarcoplasmic reticulum; SSM, subsarcolemmal mitochondria; TCA, tricarboxylic acids.

Two powerful strategies for protecting the heart against the detrimental and irreversible effects of IR myocardial injury have been pivotal to unravel the role of PTMs for cardioprotection. Ischemic preconditioning (IPC) and ischemic postconditioning (iPostC) include pharmacological treatments or mechanical maneuvers that induce brief, non-lethal episodes of ischemia and reperfusion. These are applied to the heart before, during, or even after an episode of sustained lethal myocardial ischemia [68], promoting common signaling factors recognized by cell-surface receptors, and activating protein kinase effectors and transcription factors that are directed to target executors such as mitochondria (Figure 2) [69].

### 3.1. Ischemic Preconditioning

IPC consists of brief cycles of ischemia and reperfusion applied to the heart either locally or globally before a prolonged ischemic event, to render the heart resistant to future ischemic insults [70,71]. Importantly, IPC can also be obtained by the use of pharmacological agents (pharmacological preconditioning) or by inducing ischemia to a distant organ or tissue (remote preconditioning) before the ischemic event [72]. The potential effects of IPC to protect the heart from acute myocardial infarction have been known and exploited since the pioneering work of Reimer and Murry [73].

It is well known that IPC reduces the initial burst of ROS and RNS, diminishing the deleterious effects of oxidative and nitrosative stress at reperfusion [74]. More important is the demonstration that low levels of ROS and RNS during IPC maneuvers activate redox signaling and trigger protective mechanisms or adaptive responses [49].

Numerous studies have shown that cGMP-dependent effects of NOS/NO signaling play a key role in IPC-induced cardioprotection [75]. However, while eNOS is not necessary for the early phase of IPC, the NO produced by eNOS is essential to trigger late IPC as well as the delayed activation of iNOS [76]. In this respect, NO mediates IPC-induced endothelial protection in the model of transient left anterior descending (LAD) coronary artery ligation [77] and increases cardiac contractility after global IR in isolated rat hearts [78]. The beneficial effects of IPC disappear when NOS inhibitors, including L-NAME, N^G^-nitro-L-arginine (L-NNA), and the sGC inhibitor ODQ (1H-[1,2,4]oxadiazolo-[4,3-a]quinoxalin-1-one), are used [79]. Conversely, the administration of exogenous NO mimics the protective effects of late IPC. For example, SNAP pretreatment reduces infarction in isolated rabbit hearts [80], whereas GTN infusion protects against myocardial stunning in an in vivo model of acute myocardial infarction [81]; importantly, the protection was equivalent to that observed during the late phase of IPC, and it was mediated by a PKC-dependent signaling mechanism [80].

Guo et al. (1999) demonstrated that iNOS is an essential mediator in the cardioprotection afforded by the late phase of IPC, since targeted ablation of the iNOS gene abrogates late IPC and the infarct-sparing effect in iNOS^−^^/^^−^ mice [82]. Besides, iNOS expression was upregulated in the late phase of IPC in mice cardiomyocytes and conscious rabbits [83]. IPC-induced iNOS attenuated infarct size in a rabbit model of myocardial infarction, but such effect was lost when the iNOS inhibitors dexamethasone or aminoguanidine were administrated [84]. Moreover, Wang et al. (2004) identified in conscious rabbits that the cardioprotection observed in the final stage of late IPC is mediated by nNOS in concert with cyclooxygenase-2 (COX-2), not by iNOS, since the selective inhibition of nNOS with N-propyl-l-arginine or S-ethyl N-[4-(trifluoromethyl)phenyl]isothiourea completely blocked the protection [85].

NO is also produced via the nitrate–nitrite–NO pathway [86]. The inorganic anions nitrate (NO_3_¯) and nitrite (NO_2_¯), endogenous stores of NO, are reduced through the action of a variety of proteins, including hemoglobin, molybdo-flavoproteins, mitochondrial proteins, cytochrome P450 enzymes, and NOS [87]. NO produced by this pathway has beneficial effects on endothelial dysfunction, protects against myocardial IR injury, attenuates infarct size, inhibits platelet aggregation, and modulates mitochondrial function [88]. In particular, the infusion of NO_2_¯ previous to IR generates NO by xanthine oxidoreductase activity, reduces infarct size, and improves cardiac function of isolated perfused rat hearts [89]. Similar functional results were obtained in an open chest mice model, and several NO derivatives were identified, such as S-nitrosothiols, N-nitrosamines, and iron-nitrosylated heme proteins [62]. Besides, Shiva et al. (2007) determined that NO_2_^−^-dependent protection occurs at the mitochondrial level through S-nitrosylation of CI, giving as a result the attenuation of reperfusion ROS production and aconitase inactivation that, in addition to preventing mPTP opening and cytochrome c release, reduces infarct size in mice hearts [90].

### 3.2. Ischemic Postconditioning

Ischemic postconditioning (iPostC), one of the most powerful strategies to reduce myocardial infarct size, is a mechanical maneuver in which brief cycles of reperfusion/re-occlusion of the occluded artery are applied after the ischemia and previous to long-lasting reperfusion [91]. The application of iPostC prolongs acidosis during the first minutes of reperfusion, preventing calcium (Ca^2+^) overload, ROS/RNS overproduction, and ultimately mPTP opening [92]. Such effects result from activation of the reperfusion injury survival kinase (RISK) family, which includes the extracellular signal-regulated kinase 1/2 (ERK1/2), phosphatidylinositol 3-kinase (PI3K/AKT), and protein kinase C (PKC) [93], but also from the induction of the classical NO/sGC/cGMP signaling pathway [94] and the maintenance of redox homeostasis by preserving nuclear factor E-2 related factor 2 (Nrf2) activation [95] via the activation of PKC and ERK1/2 by H_2_O_2_ [96]. Of note, iPostC maintains redox homeostasis in reperfused hearts, as evidenced by the administration of reductive agents such as N-acetyl cysteine [97], 2-mercaptopropionylglicyne [98], and ascorbic acid [96], which abolish the iPostC-associated cardiac protection.

The cardioprotection associated with iPostC has also been related to decreased nitrotyrosine levels in myocardial proteins and increased SNO [99]. Certain metabolic conditions such as hyperglycemia induce myocardial overexpression of iNOS, abolishing the protective effect of iPostC in correlation with nitrotyrosine increase due to ONOO^−^ formation [100]. Interestingly, the administration of pharmacological NO-donors induces cardioprotection in a similar way to that exerted by iPostC [66].

## 4. Reversible Oxidative Post-Translational Modifications of Cardiac Proteins during Myocardial Reperfusion and Cardioprotection

Cysteine residues are modified due to the chemical reactivity of the thiol group. The nucleophile thiolate, the deprotonated form of the thiol group, reacts with oxidants and electrophilic molecules producing the reversible PTMs, including sulfenic acid, S-nitrosothiol, disulfides, and S-glutathionylation (SG), which regulate protein activity, stability, compartmentalization, and protein–protein interactions [32]. On the other hand, irreversible redox PTM including 3-nitrotyrosine, protein carbonyl and sulfonic acids are related to protein aggregation and degradation [101].

### 4.1. S-Nitrosylation

The biochemistry of SNO is reviewed in detail by Fernando et al. (2019) [102]. Broadly speaking, several mechanisms of SNO formation have been described, but the most important one occurs after oxidative reactions. Cellular homeostasis of SNO is regulatedby at least two mechanisms: (1) transnitrosylation, the reversible transfer of an NO group from one cysteine residue to another, which propagates SNO-based signals; and (2) denitrosylation, the reverse reaction of SNO that removes an thiol-NO group from nitrosylated proteins [10]. Experimentally, about 4000 S-nitrosylated sites associated with more than 3000 proteins have been identified and classified in terms of their structural features, their functionality, their relevance in disease, and the regulatory networks of S-nitrosylated proteins [10]. In the myocardium, Kohr et al. (2011) characterized both the SNO proteome and its potential sites of action, highlighting key proteins involved in myocardial contraction (e.g., myosin light chain 3), metabolism (e.g., lipid metabolism, cell death), and cellular signaling (e.g., caveolin-3) [103]. Besides, this group identified approximately 177 S-nitrosylated proteins that contribute to gender-dependent cardioprotection [104]. In this regard, it has been documented that under basal conditions, male and female hearts contained about 4 pmol of S-nitrosylated proteins per milligram of protein [105].

Hypo- or hyper-SNO contributes to the pathophysiology of several diseases, including IR [106]. To mention some examples, KO mice of the enzyme GSNO reductase showed reduced infarct size and preserved cardiac function associated with SNO of the hypoxia-inducible factor 1 alpha (HIF-1α) and with upregulation of the vascular endothelial growth factor (VEGF), a mediator of cardiac angiogenesis [107]. Meanwhile, nNOS^−^^/^^−^ mice show hypo-S-nitrosylation of the ryanodine receptor type 2 (RyR2) and loss of diastolic Ca^2+^ that concurs with arrhythmias and sudden death [108]. Furthermore, eNOS activity increase in the VSMCs and endothelium correlates with NO-induced covalent modifications in sulfhydryl-containing proteins [109]. In this regard, SNO of Cys^3635^ at the ryanodine receptor underscores the mechanism of calmodulin-dependent receptor regulation by NO in the skeletal muscle [110].

On the other hand, SNO shields cysteine residues against potential oxidative damage from ROS and prevents from further oxidative modifications in proteins that may alter their structure and function [111,112]. Even more, Sun et al. (2013) suggested that the IPC-induced protective effect of NO is related primarily to SNO signaling, rather than to the activity of the sGC/cGMP/PKG pathway [113]. IPC increased SNO content in isolated mitochondria from rat hearts subjected to IR [114], improving cardiac function and reducing infarct size in Langendorff perfused mice hearts. Such effects were similar to those observed when hearts were pretreated with GSNO [60].

In addition, SNO abrogates protein oxidation in isolated hearts subjected to global ischemia [115]. IPC-induced SNO prevents the degradation of TRIM72, a membrane repair protein, and reduces infarct size in ischemic hearts [116]. IPC also increased SNO content in isolated mitochondria from rat hearts subjected to IR [114,117]. In this respect, it was demonstrated that eNOS/NO/SNO signaling is driven through caveolae to mitochondria and that disruption of this structure with the cholesterol sequestering agent, methyl–β–cyclodextrin (MβCD), inhibits SNO and cardioprotection [118]. Another pathway described for trans-S-nitrosylation from the cytosol to mitochondria is mediated by the glycolytic enzyme glyceraldehyde-3-phosphate dehydrogenase (GAPDH). S-nitrosylated GAPDH (SNO-GAPDH) enters into the mitochondrial matrix where it interacts with mitochondrial proteins and directly transfers the NO moiety to a recipient protein and promotes SNO [119].

Recently, it was described that cardiac subsarcolemmal mitochondria [120] isolated from Langendorff perfused mouse hearts are the preferential target for NO/SNO signaling, and that their proteins are more susceptible to be S-nitrosylated in comparison with interfibrillar mitochondria (IFM) [121]. Pioneering works showed that mitochondrial connexin 43 (Cx43), a gap junction protein exclusively localized in SSM, was involved in infarct size reduction by IPC [122]. Recently, it was discovered that IPC-increased nitrosation of Cx43 and regulates mPTP [123]. Chouchani et al. (2013) demonstrated that IPC stimulates the reversible S-nitrosation of Cys^39^ on the ND3 subunit of mitochondrial CI and showed evidence that this modification mediates the inhibition of CI activity, functioning as a molecular switch in the regulation of mitochondrial ROS production and cell death, thereby limiting IR damage [124]. Some of the IPC-induced SNO proteins include sarcoplasmic reticulum Ca^2+^-ATPase (SERCA2a), F_1_F_0_-ATPase, NADH:ubiquinone oxidoreductase (CI), creatine kinase, hexokinase-1, aldehyde dehydrogenase, acyl-CoA dehydrogenase, citrate synthase, aconitase, α-ketoglutarate dehydrogenase, malate dehydrogenase, aspartate aminotransferase, and Trx [60,115,117].

Equally important, the pre-ischemic administration of S-nitroso-2-mercaptopropionyl-glycine (SNO-MPG) protects perfused hearts and isolated cardiomyocytes from IR injury by inhibiting CI activity via S-nitrosation and inhibits cellular respiration. In good accordance, mitochondria isolated from SNO-MPG-treated hearts subjected to ischemia showed improved tolerance to Ca^2+^ overload and reduced ROS levels [125]. Comparable results were obtained in a LAD occlusion model [126]. Furthermore, pharmacological preconditioning with the adenosine A1 receptor agonist N6-cyclohexyl adenosine (CHA) leads to activation of the PI3K/AKT/eNOS signaling cascade, with the consequent increment of SNO protein levels and enhanced post-ischemic functional recovery in both male and female hearts [127]. Besides, it has been described that the mechanism of action and protection of statins are related to SNO. Atar et al. (2006) described that atorvastatin reduces infarct size after increasing iNOS and COX-2 activity through SNO in rats [128]. Although NO treatments have been widely used as adjuncts to reperfusion in acute myocardial infarction [129], the value of these interventions in the clinic is not fully proven, as different confounding scenarios and co-morbidities prevail in clinical settings. However, it is clear that among other PTMs, SNO is at the forefront of the redox signaling paradigm in cardiac protection.

### 4.2. S-Glutathionylation

S-Glutathionylation (SG) is a reversible protein modification that occurs through the addition of a donor of glutathione to thiolate anions of cysteines in target proteins, which alters their molecular mass, charge, structure, and function and is directly influenced by redox homeostasis [130]. SG is catalyzed by glutathione S-transferase and the reverse reaction by glutaredoxin; therefore, redox signaling-dependent events are controlled by reduced GSH content, the GSH/oxidized glutathione (GSSG) ratio, and the cellular redox state. It is known that SG of mitochondrial CI affects ATP production in the cell [131], and that the activities of both the transcription factor nuclear factor-kappa B (NF-kappa B) and kinase AKT decreased when these proteins are glutathionylated [132]. On the other hand, SG activates SERCA [133], as well as the RyR1 in skeletal muscle cells [134], whereas SG promotes eNOS uncoupling, favoring superoxide anion production [135].

There are few reports on the effect or the prevalence of SG in the cardioprotective context. It has been reported, for example, that GSNO treatment, the major endogenous S-nitrosyl donor, provides similar cardioprotective effects to IPC against IR [60] and that pharmacological preconditioning with apocynin, an NADPH oxidase inhibitor, prevents SG of RyR2, modulating its activation and calcium release in Langendorff perfused rat hearts [136]. Moreover, tachycardia-induced preconditioning promotes SG of RyR2 [137], but also induces SG of cyclophilin-D, which is a crutial component of mPTP, preventing pore opening and cardiomyocyte apoptosis [138].

### 4.3. S-Sulfhydration

Protein cysteine residues can also be modified by hydrogen sulfide (H_2_S) yielding S-sulfhydration, a redox PTM that confers cardioprotection by regulating angiogenesis, inflammation, oxidative stress, and apoptosis [139,140]. The effect of H_2_S is related with eNOS activation, NO-signaling, and SNO [141].

The protective role of H_2_S against myocardial reperfusion injury is related to preservation of mitochondrial function [142]. Recently, it was reported that H_2_S-mediated cardioprotection in preconditioned aged hearts is associated with upregulation of HIF-1α and Nrf2 signaling pathways [143]. H_2_S also induces a preconditioning-like effect, preventing reperfusion injury in diabetic rat hearts by decreasing infarct size and preventing mitochondrial dysfunction via PI3K/GSK3β signaling [144].

Furthermore, exogenous H_2_S restores iPostC-associated cardioprotection in aged cardiomyocytes, via upregulation of the heparin-binding epidermal growth factor (HB-EGF)/ EGF receptor signaling and the activation of ERK1/2, PI3K/AKT, and glycogen synthase kinase-3β (GSK3β) [145]. Indeed, both NO and H_2_S lead to a synergistic effect to protect the heart, reducing myocardial infarct size via the increase in SNO [139]. Alternatively, it is worth to mention that H_2_S preconditioning in gastric epithelial cells induces Keap-1 S-sulfhydration, which promotes Keap1/Nrf2 disassociation and Nrf2 activation, giving as a result that H_2_S prevents I/R-induced oxidative stress [146].

### 4.4. S-Sulfenylation

Cysteine residues can be oxidized to yield sulfenic acid, another redox switch that regulates protein function. Sulfenic acid can be further oxidized to higher-oxidation-state species that are regarded either as reversible or irreversible. On one hand, sulfenic acid reaction with GSH produces S-glutathionylated cysteines [147], whereas sulfonic or sulfinic acid are irreversible modifications and markers of oxidative damage. Cysteine S-sulfenylation has been identified as a redox sensor in an increasing number of proteins. For example, it favors the degradation of the voltage-gated potassium channel Kv1.5 in hearts from patients with chronic atrial fibrillation [148]. Due to its short life, S-sulfenylation has been proposed as an intermediate modification that induces conformational changes in proteins, enabling its adaptation to myocardial redox environment changes [149].

## 5. Signal Delivering: The Role of Caveolae and Caveolins

Caveolae (50–100 nm) are the most abundant and striking features of the plasma membrane (PM) in many cell types. They are bubble-like invaginations composed of specific proteins and lipids [150], located in the plane of the PM or as detached vesicles. They can fuse and form grape-like or tubule-like structures with sizes significantly larger than 100 nm [151]. Caveolae occupy up to 50% of the surface of some mammalian cells, such as skeletal muscle, and adopt a uniform shape resembling an omega [152]. The caveola is laterally oriented due to the coordinated action of integral membrane proteins (caveolin) together with peripheral membrane proteins (cavin) that integrate the caveolar bulb [150]. Other caveolar components include the EH domain-containing protein 2 (EHD2, a large ATPase located in the caveolar neck), PACSIN2, and the tyrosine kinase-like orphan receptor 1 (ROR1) that binds caveolin-1 and cavin-1 to facilitate caveolae formation [150]. So far, three caveolin isoforms have been identified, but Cav-1 and 3 are the most relevant in cardiac muscle under both physiological and pathological conditions [153,154].

These structures are related to the regulation and spatial compartmentalization of various signaling cascades, such as eNOS [154], serving as platforms to integrate the activity and/or localization of these molecules and their effectors [155]. Such molecules bind to Cav-1 through its caveolin scaffolding domain (CSD) and concentrate in caveola lips rafts where they are activated or deactivated [156]. In this regard, Garcia-Cardeña et al. (1996) showed that targeted mutagenesis on the caveolin binding motif in eNOS inhibits its activity. This study demonstrated for the first time the role of Cav-1 as an endogenous regulator of NOS and, in general, as a regulator of signal transduction [157].

In the context of IR, Ballard-Croft et al. (2006) described that early reperfusion reduces Cav-3 expression and that disruption of caveolae attenuates cardiomyocyte protection during ischemia [158]. Cav-3^−^^/^^−^ rodents are refractive to isoflurane-induced cardioprotection [159], supporting the participation of caveolin in myocardial protection. On the other hand, Cav-3 overexpression increases caveolae formation, reduces infarct size, and boosts phosphorylation of kinases involved in cardioprotection. Thus, Cav-3 can protect from IR to an extent comparable to that induced by preconditioning [160]. Indeed, subcellular redistribution of caveolin-3 is a key mechanism in cardiac protection. Data from our group show that ERK1/2 is associated with Cav-3-enriched vesicles and delivered to mitochondria in iPostC hearts [161]. Subsequently, García-Niño et al. (2017) confirmed that caveolae formation is related to Cav-3 upregulation and ERK1/2 activation in SSM and IFM mitochondria and hence with cardioprotection [162]. Other caveolae-associated signaling proteins include AKT, GSK-3β, and some PKC isoforms [161], not to mention their association with eNOS in SSM mitochondria from iPostC hearts [121].

Sun et al. (2012) reported that caveolar disruptionwith MβCD blocks cardioprotective eNOS/NO/SNO signaling to mitochondria. Furthermore, it diminishes the levels of SNO proteins and inhibits preconditioning-induced protection [118]. Additionally, this group reported that subsarcolemmal mitochondria are preferential targets of modifications by SNO derived from sarcolemmal signaling in IPC-mediated cardioprotection, due to the proximity of this type of mitochondria to the sarcolemmal caveolae [121].

On the other hand, in hearts from wild-type female mice treated with isoproterenol before IR, Sun et al. (2006) reported a dramatic increase in S-nitrosylation content, specifically in the L-type Ca^2+^ channel, suggesting an elevation in eNOS levels; the authors confirmed that this augment is due to eNOS association with Cav-3 in female hearts. Interestingly, the hearts from eNOS^−^^/^^−^ mice do not show differences in SNO levels [105].

## 6. Post-Translational Modifications in Mitochondria: The Ultimate Cardioprotection Target

PTMs are a primary mechanism for mitochondria to communicate with the rest of the cell [163]. The mitochondrial proteome is subjected to multiple reversible PTMs that modify relevant functions as energy generation, cell death regulation, Ca^2+^ transport, and production/metabolism of ROS [164]. SNO has been documented as the more common redox-based PTMs occurring in mitochondrial proteins, for example the components of the mPTP and the respiratory chain complexes. It is also known that the nitrosative agent GSNO leads to S-nitrosylation of the ATPase, the adenine nucleotide translocase (ANT), and the voltage-dependent anion channel (VDAC) when administrated to isolated mitochondria. These proteins have been pointed out as major components of the mPTP [165], a non-specific mega channel, that induces depolarization of the mitochondrial inner membrane leading to ATP depletion and enhanced colloidal osmotic pressure in the mitochondrial matrix, producing matrix swelling and rupture of the mitochondrial outer membrane [166]. SNO of ANT and VDAC correlates with Ca^2+^-dependent swelling of isolated rat heart mitochondria and with cyclosporine A-sensitive-cytochrome c release in perfused hearts [167]. In addition, SNO of Cys^203^ in cyclophilin D (CypD), the main mPTP regulator, has been suggested to have a similar effect on mPTP opening to that observed with CypD deletion [168].

The mPTP is considered the end target in cardioprotection; however, to date it is not completely clear whether SNO plays a role in physiological mPTP regulation. In this sense, it has been shown that NO signaling prevents reperfusion damage by both cGMP-dependent and SNO pathways [113], reporting increased SNO levels after blocking the sGC/cGMP/PKG signaling pathway in correlation with IPC-mediated cardioprotection; whereas our group found the partial recovery of heart function and lower rates of mPTP opening when the NO donor (Z)-1-[N-(2-aminoethyl)-N-(2-ammonioethyl)amino] diazen-1-um-1,2-diolate (DETA-NO) was administrated to postconditioned hearts in which sGC was inhibited [94].

Although these results suggest a major role of the SNO pathway in the observed cardioprotection, SNO could modify other mitochondrial functions or even extramitochondrial targets that contribute to regulate mPTP opening. For example, the activation of calpain-1, a Ca^2+^-dependent cysteine protease, is related to mitochondrial-dependent apoptosis and with necrotic processes driven by calcium overload and mPTP opening. We recently found that increased SNO in the small subunit of calpain-1 induced by S-nitroso-N-acetylpenicillamine concurs with diminished cardiac calpain activity in postconditioned hearts [66]; whereas Thompson et al. (2016) demonstrated that the calpain inhibitor improves mitochondrial Ca^2+^ retention capacity from ischemic/reperfused hearts and therefore prevents mPTP opening [169].

On the other hand, S-nitrosothiols and IPC produce the reversible inhibition of CIby SNO of its 75 kDa subunit in heart mitochondria [114] and protects perfused hearts from IR injury. It is suggested that such an effect is related to reduction of ROS production by slowing the re-entry of electrons to the respiratory chain during early reperfusion [125], a mechanism also proposed in iPostC-mediated cardioprotection [170]. Years later, Chouchani et al. (2013) demonstrated that Cys^39^ within the ND3 subunit of CI is S-nitrosylated by the mitochondrial targeted S-nitrosothiol, MitoSNO, inhibiting ROS production and diminishing IR injury [124].

Mitochondrial CI is also a target of thiol-modification SG, but the physiological meaning of such PTMs is far from being understood. Even though the complex activity is strongly inhibited in cardiac mitochondria under conditions of oxidative stress [171], glutathionylation reactions also occur when GSH/GSSG is high [172]. Even more, different studies have found that SG at CI can increase or decrease O_2_^•−^ formation [173]. A relevant finding is that the Ndusf1 subunit and other mitochondrial proteins are reversibly S-glutathionylated by glutaredoxin-2 (Grx2) [174], which opens the exciting possibility that this protein could be a bona fide redox sensor/regulator of mitochondrial ROS production and redox variations.

Conversely, it has been observed that SG of the FAD-binding subunit in Complex II activates the enzyme in vivo, and that under stress conditions deglutathionylation of the complex concurs with its inactivation and increased ROS production in hearts subjected to IR [175].

The mitochondrial matrix is a highly reducing environment that contains high levels of GSH and exposed protein thiols. These unique biochemical properties and redox conditions in mitochondria favor SG. Even more, ROS enhances such reactions by oxidizing cysteine thiols or increasing GSSG levels [172]; therefore, it is not surprising that many mitochondrial proteins are susceptible to be S-glutathionylated depending on ROS levels, cell type, or the physiopathological state. Aconitase [176], α-ketoglutarate dehydrogenase [177], Complex IV and Complex V [178], uncoupling protein 2 (UCP2) [179], the ANT [180], mitofusin 1 and 2 [181], and superoxide dismutase [182] are examples of such proteins. However, no reports have addressed the impact that the glutathionylated state of these proteins has on mitochondrial function and cell signaling in the context of cardioprotection.

## 7. Therapeutic Strategies: Antioxidants or Nitric Oxide Donors

**Figure 3:**
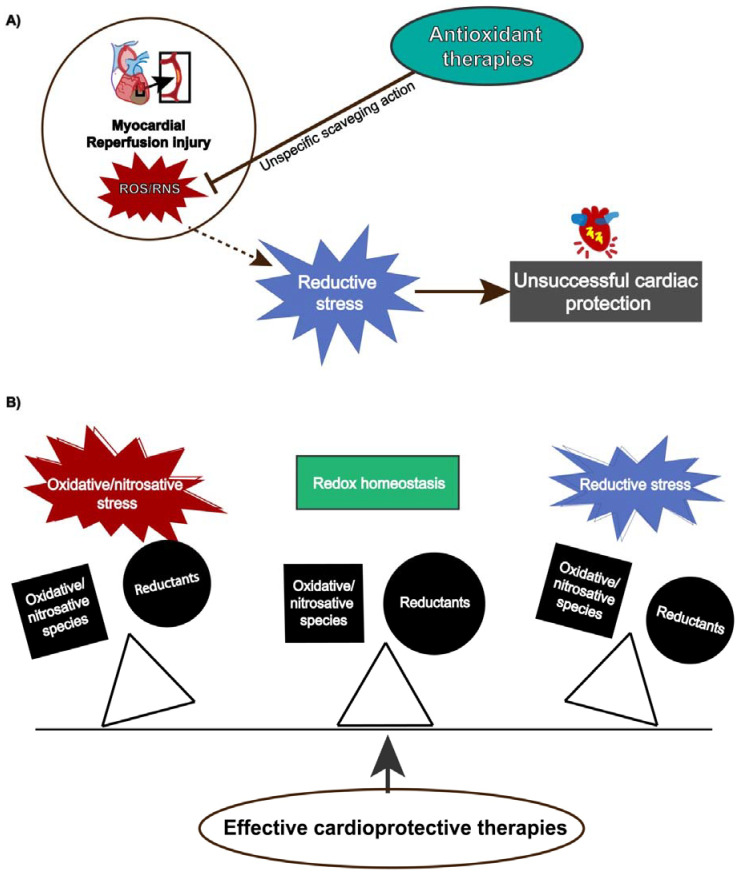
(**A**) Reductive stress induced by antioxidants attenuates cardiac protection. (**B**) Effective cardioprotective strategies preserves redox balance and prevents oxidative/nitrosative or reductive stress.

Although reperfusion injury is closely associated with ROS overproduction, protective strategies using antioxidants have not shown absolute efficacy [183] because ROS/RNS also exert physiological functions depending on their concentration and temporal action. Antioxidant therapies might produce pro-reductive conditions, which also induces cellular damage. This condition, termed reductive stress, has gained notoriety, contributing to understanding the paramount role of redox homeostasis and that any unbalance either towards oxidative or reductive stress can lead to detrimental cellular effects (Figure 3).

### 7.1. Reductive Conditions, an Alternative Therapy against Oxidative Stress?

The search for cardiac protective strategies that maintain reductive conditions without reaching reductive stress underlies many unsuccessful treatments of cardiac pathologies involving oxidative stress. It is known that cellular pro-oxidative status leads to the activation of transcription factors such as Nrf2, which constitutive activation may produce reductive stress in the myocardium, inducing deregulation of the antioxidant and metabolic response, inhibiting detoxifying enzymes, alterating the unfolded protein response (UPR), and overall of redox signaling. Nrf2, the master regulator of the antioxidant system in the cell, is regulated by its repressor Keap1 [184], which acts as a redox sensor. Oxidation of its reactive cysteine residues (on positions 151, 273, and 288) promotes the dissociation of Nrf2 and its nuclear accumulation. There, it heterodimerizes with transcriptional cofactors to promote the expression of antioxidant and electrophilic response elements (ARE/EpRE)-containing genes [93]. Once the redox state is re-established in the cell, Nrf2 is repressed by Keap1. Therefore, any condition that alters Nrf2 regulation might induce reductive stress.

Heart-specific genetic mouse models constitutively expressing active Nrf2 (caNrf2) have been employed to induce pro-reductive and reductive stress [185]. The detrimental effects triggered by caNrf2-driven reductive stress include pathological cardiac remodeling [120,185] along with cytoskeletal alterations, deregulation of Ca^2+^ handling, and alteration of the contractile apparatus [120]. Recently, it has been reported that the mesencephalic astrocyte-derived neurotrophic factor (MANF), an endoplasmic reticulum (ER) resident, exerts an adaptive response to reductive stress, improving ER protein folding and cardiac myocyte viability during IR [186].

Reductive stress may lead to detrimental cellular effects. Therefore, we should be aware that antioxidant therapies are not innocuous, since unspecific ROS removal may affect essential processes such as metabolism, Ca^2+^ homeostasis, vascular function, inflammation, and a wide range of signaling pathways that affect the cardiovascular system. So far, strategies to prevent reperfusion injury might consider Nrf2 regulation as a key target.

### 7.2. Nitric Oxide Donors

It has been well established that nitric oxide donors can induce cardiac protection. DETA-NO protects from reperfusion injury, enhancing NO levels associated with caveolin-3 and regulating the extracellular matrix metalloproteinase (MMP) inducer, involved in MMP2 and MMP9 expression [187]. In addition, the nitric oxide donor 5-phenyloxyphenyl-5-aminoalkyl nitrate barbiturate inhibits MMP-2 and prevents reperfusion injury in both ex vivo and in vitro models [188]. The mechanisms behind the cardioprotective effect of SNAP in isolated rabbit hearts are reported to be independent of PKG and downstream of AKT and ERK signaling [189]. This NO donor along with sodium nitroprusside and 3-morpholinosydnonimine also regulate iron metabolism and activate redox metabolism, triggering cardioprotection in rat hearts [190]. Although it is clear that nitric oxide induces cardioprotective signaling and diminishes reperfusion damage, it should be highlighted that such an effect depends on its concentration and on the redox environment that, if unbalanced towards oxidative conditions, could lead to RNS production.

## 8. Conclusions

Substantial progress has been made in understanding how redox PTMs modulate important cellular functions in cardiac physiopathology, highlighting the paramount role of redox homeostasis. NO and its derivatives are fundamental in IPC- and iPostC-induced cardioprotection, whereas reversible SNO of mitochondrial proteins is crucial for preventing structural and functional changes of ROS/RNS-modified proteins. Downstream signaling pathways that reduce Ca^2+^ overload, prevent mPTP opening, and attenuate cell death point out the central role of mitochondria in both cardioprotective maneuvers. The intricate network of mitochondrial SNO proteins involved in these mechanisms requires further studies to elucidate the impact of such post-translational modifications on mitophagy, fusion/fission processes, and proteasomal degradation in the setting of acute myocardial infarction. In addition, recognition of the relevance of caveolae on NO signal delivery to mitochondria further addresses the physiological and pathological importance of these modifications in heart disease and may be a potential therapeutic target for preventing and/or treating reperfusion damage.
